# Alopecia Areata After Initiation of Secukinumab Therapy for Plaque Psoriasis

**DOI:** 10.7759/cureus.38986

**Published:** 2023-05-13

**Authors:** Esther Choi, Olivia Thomson, David Smith

**Affiliations:** 1 Dermatology, Washington State University, Spokane, USA; 2 Dermatology, Washington State University, Everett, USA; 3 Dermatology, The Everett Clinic, Everett, USA

**Keywords:** biologic therapies, il-17 inhibitor therapy, psoriasis, secukinumab, alopecia areata

## Abstract

The development of psoriasis and alopecia areata (AA) is multifactorial. The interleukin-17 (IL-17) cytokine is believed to be associated with the pathophysiology of both diseases. This case report demonstrates a 64-year-old female patient who experienced a new onset of AA after the initiation of IL-17A inhibitor, secukinumab, for the treatment of her psoriasis. To our knowledge, there are only three case reports specifically discussing IL-17A inhibitors and AA. This case report highlights a potential rare but significant side effect of IL-17A inhibitors.

## Introduction

The development of psoriasis is multifactorial; it is a consequence of genetic predisposition, environmental triggers, and autoimmune pathogenic traits [1]. Alopecia areata (AA) also demonstrates a similar pathogenic triad to psoriasis, and both psoriasis and AA have been found to co-exist together [2]. The disturbances in the immune responses have been of great interest for pharmacological development against these conditions. In particular, studies have found that interleukin-17 (IL-17) and interleukin-23 (IL-23) are key drivers for the pathogenesis of psoriasis [1,3], and the IL-17 cytokine family is also believed to induce AA [4]. Studies have shown an elevation of IL-17 in the area of alopecia as well as in the blood [5]. Therefore, it would seem the new biologic secukinumab, an IL-17A inhibitor, would treat both psoriasis and AA. However, paradoxically, we present a case report demonstrating new AA onset after initiation of secukinumab to treat plaque psoriasis.

## Case presentation

A 64-year-old Caucasian female presented with a history of chronic plaque psoriasis. Her past medical history was significant for treated chronic hepatitis C, 30 pack-year history of tobacco use, chronic obstructive pulmonary disease (stable on budesonide/formoterol and albuterol since 2017), hypertension, and sleep apnea.

**Figure 1:**
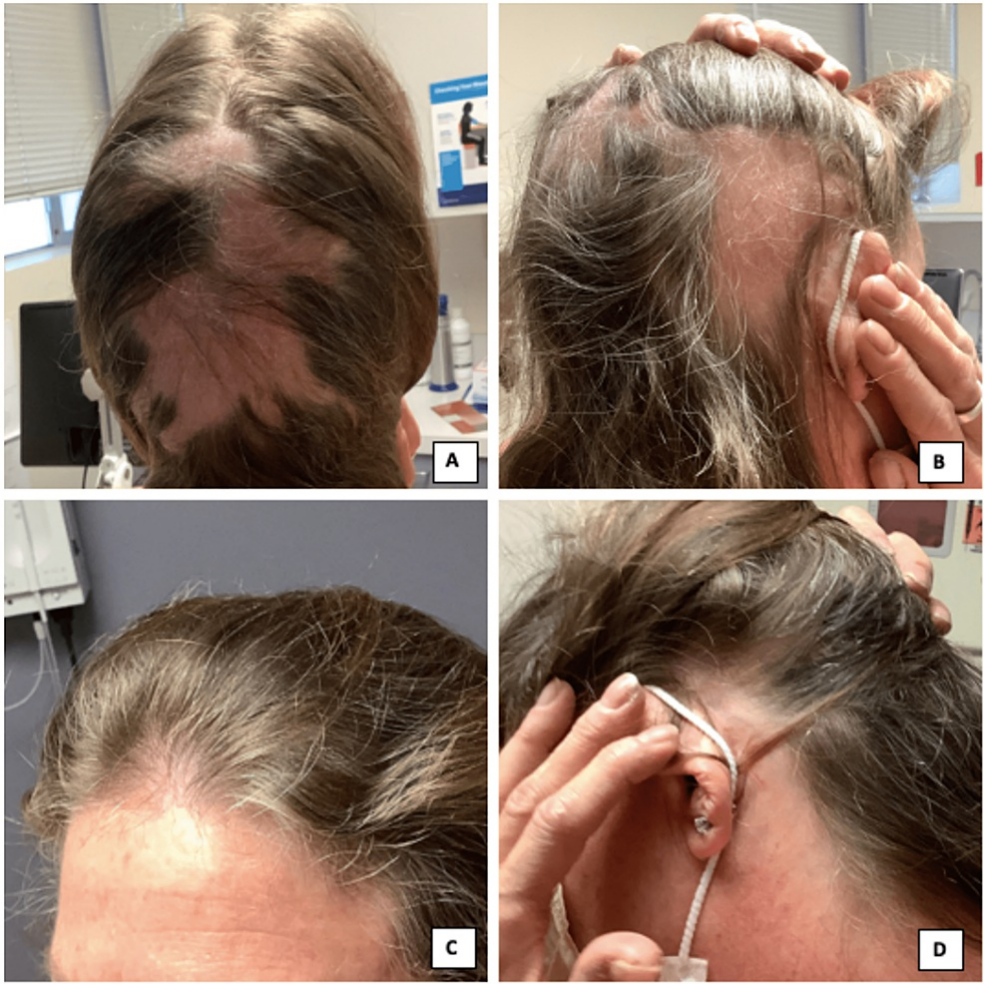
Extensive hair loss on posterior and vertex scalp (A), right temporal scalp (B), frontal scalp (C), and left temporal scalp (D) one month after initiation of secukinumab.

**Figure 2:**
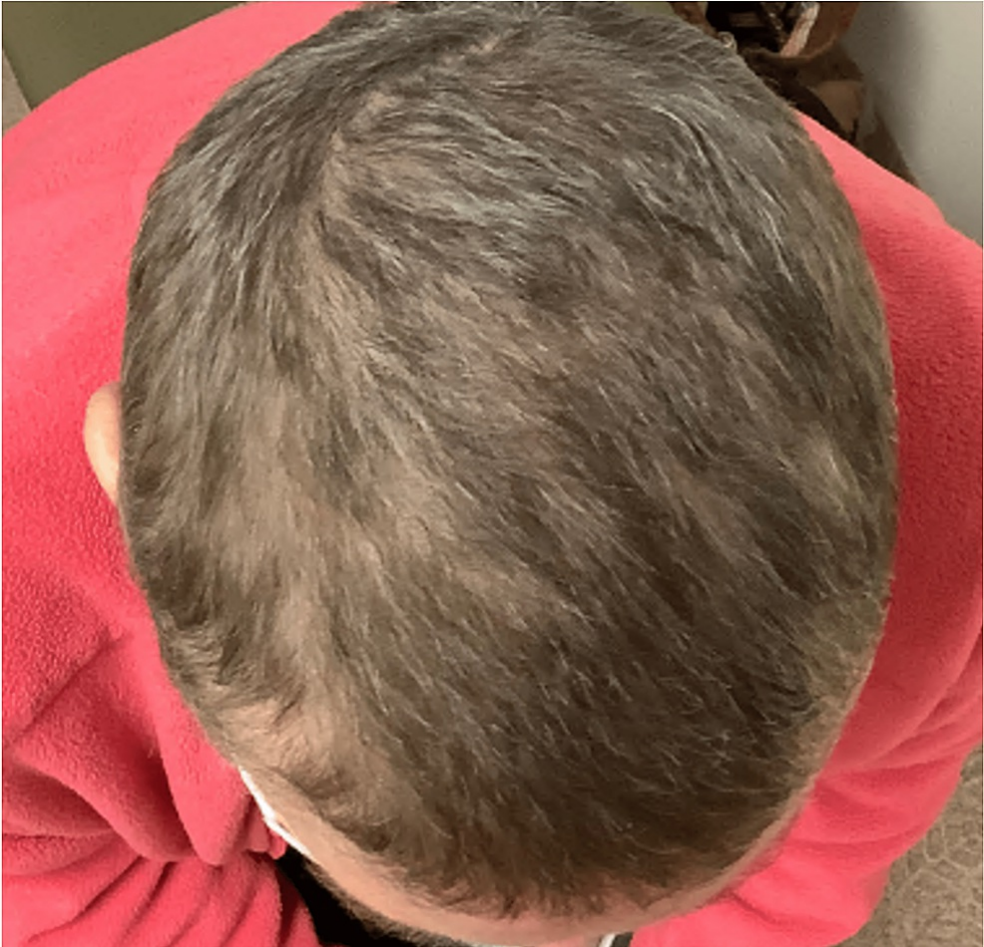
Hair regrowth on the vertex, mid-scalp, and front scalp three months after discontinuing secukinumab.

**Figure 3:**
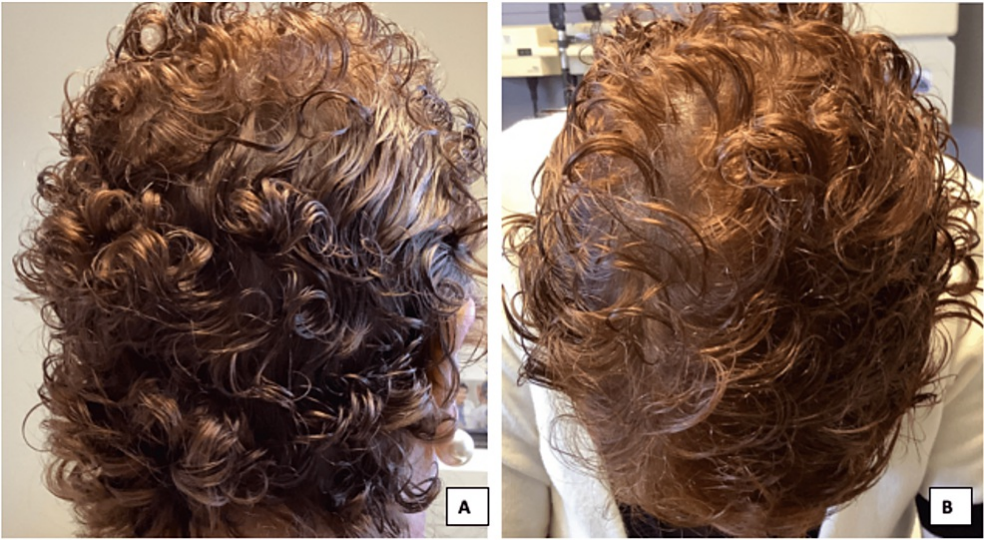
Complete hair regrowth to its original fullness on the posterior scalp (A) and crown of scalp (B), eight months after discontinuing secukinumab.

The initial physical exam demonstrated hyperkeratotic red scaly plaques on the palms, fingers, toes, thighs, and shins. Her condition was previously treated with topical triamcinolone for two years from 2018 to 2020 and narrow-band ultraviolet B phototherapy from November 2018 to January 2019 with minimal improvement. The patient was also treated with acitretin during this time, but she developed flushing and gastrointestinal upset prompting the discontinuation of this therapy. Her primary care physician initiated treatment with adalimumab (unknown dose) in October 2019, which was complicated by the development of generalized pustular psoriasis. Adalimumab was discontinued in May 2020, and she was transitioned to secukinumab 300 mg subcutaneous injection in June 2020 with resolution of pustular psoriasis within one month. However, six weeks after the initiation of secukinumab, the patient called to report a new onset of hair loss. She was seen one week later, and her examination was notable for significant progression of her psoriasis with new onset involvement of her arms and trunk. Additionally, she developed large, confluent, well-demarcated patches of alopecia in the posterior and vertex of her scalp with no other areas of alopecia noted on examination (Figure 1). A biopsy of the patient’s scalp was not obtained as the clinical findings were consistent with AA. The patient’s serum thyroid stimulating hormone, free T4, and ferritin levels were within normal limits. Secukinumab was discontinued after two months of use and was substituted with the IL-23 inhibitor, guselkumab (100 mg/mL every eight weeks). Her psoriasis responded well to guselkumab, though she continued to have active involvement of her dorsal hands and feet. Within four weeks after discontinuation of the secukinumab and two weeks after the initiation of guselkumab, there was regrowth of hair within the patches of alopecia on her scalp. Three months after discontinuation of secukinumab, her scalp hair had regrown (Figure 2), and then to its complete, original fullness by eight months (Figure 3). It should be noted the patient changed her hairstyle multiple times within the timeframe of this case report. 

## Discussion

Treatment of psoriasis has drastically changed since the mid-20th century. Specifically, the introduction of biologic therapies in 2004 made a significant impact on patients with psoriasis refractory to traditional therapies [6]. While serious infections are the most significant adverse effect of biologics, clinical studies have shown a favorable safety profile. A safety analysis of 10 phase II and III studies for secukinumab was conducted, which showed nasopharyngitis, headaches, and upper respiratory infections to be the most common side effects in the first 12 weeks of use [5]. Here, we present a case where AA may also be an adverse effect associated with the IL-17A inhibitor secukinumab.

The IL-17A cytokine is associated with inflammation, which is believed to have a key role in both psoriasis and AA [4,7]. Interleukin 17 inhibitors were hoped to be a potential option for treating alopecia [7]. However, a recent double-blinded, randomized clinical trial showed no significant benefit for treating AA with secukinumab [8]. Additionally, our patient exhibited a new onset of AA shortly following the initiation of secukinumab and resolution upon switching to guselkumab, an IL-23 inhibitor. The timing is suggestive of a possible association between secukinumab and AA, further supporting the association of the IL-17 pathway and AA. However, we did not rechallenge our patient with secukinumab to confirm whether AA would recur. 

The underlying mechanism of AA upon IL-17A inhibitor exposure remains to be elucidated. To our knowledge, there are only three case reports specifically discussing IL-17A inhibitors and AA. Interestingly, the patients in these reports developed hair loss after 2, 13, and 24 months succeeding the initiation of the biologic [9,10]. Our patient developed AA more rapidly than what was reported in these case reports. It is possible that her development of AA was due to her exposure to adalimumab, which has been previously reported [11]. Our patient was taking adalimumab for seven months prior to the initiation of secukinumab between October 2019 and May 2020. However, the rapid onset of hair loss following the initiation of secukinumab and prompt resolution after discontinuation suggest a direct reaction to this therapy. Alternatively, the overlap of two biologics within a short time frame could have triggered the patient’s new onset of AA. Additionally, although the patient did not report any significant life stressors before or during secukinumab therapy, recall bias regarding life stressors cannot be completely ruled out as there is an established link between stressors and AA [12]. 

## Conclusions

In this case report, we describe a patient who developed AA after initiation of secukinumab for treatment of psoriasis with prompt recovery upon cessation of the biologic. This case report highlights the necessity of continued observation and documentation of potential adverse effects of new-era biologic therapies. Drug reactions such as AA negatively impact patients’ quality of life and psychological health. Providers should be aware of AA as a potential adverse event when they are prescribing secukinumab or other IL-17A inhibitors for the treatment of psoriasis. 
